# Association of plasma and urine viscosity with cardiometabolic risk factors and oxidative status. A pilot study in subjects with abdominal obesity

**DOI:** 10.1371/journal.pone.0204075

**Published:** 2018-10-09

**Authors:** Beatriz Herranz, María Dolores Álvarez, Jara Pérez-Jiménez

**Affiliations:** 1 Dpt. Characterization, Quality and Safety, Institute of Food Science, Technology and Nutrition (ICTAN-CSIC), Madrid, Spain; 2 Dpt. Metabolism and Nutrition, Institute of Food Science, Technology and Nutrition (ICTAN-CSIC), Madrid, Spain; Stellenbosch University, SOUTH AFRICA

## Abstract

There is increasing interest in the search for accurate, repeatable and widely applicable clinical biomarkers for the early detection of cardiometabolic alterations and oxidative status. Viscosity is a promising tool in that sense, although most studies have used simple viscosimeters, providing limited information, and have not considered oxidative status. The aim of this study was to assess whether viscosity determinations were associated with cardiometabolic and oxidative status in subjects at a primary stage of cardiometabolic risk. A pilot study (*n* = 20) was conducted in subjects with abdominal obesity, determining urine and plasma viscosity with a rotational rheometer at different shear rates (10000–1000 s^–1^ in plasma and 1000–50 s^–1^ in urine). Simple regression showed that urine viscosity was significantly (*p*< 0.05) associated with markers of oxidative status, and plasma viscosity with blood glucose. Categorical Principal Component Analysis plots showed that urine viscosity measurements at different shear rates clustered in three groups (low, intermediate and high shear rates) were selectively associated with uric acid, polyphenols and antioxidant capacity respectively. Plasma viscosity did not seem to be a relevant clinical marker in subjects with abdominal obesity. Therefore, urine viscosity could potentially serve as a complimentary marker in the evaluation of oxidative status.

## Introduction

Non-communicable diseases such as cardiometabolic pathologies are among the most frequent causes of death, a trend which is expected to increase in coming years [1]. These pathologies, or previous high risk situations, are commonly diagnosed based on a combination of biochemical and anthropometric parameters; such is the case of metabolic syndrome, where several criteria must be simultaneously fulfilled [2] implying several determinations. Besides, oxidative status is known to be impaired in cardiometabolic pathologies [3]. Nevertheless, its evaluation also comprises different measurements, from the activity of antioxidant enzymes to concentrations of specific oxidized biomolecules [4], some of them difficult to apply routinely to large sample batches and to be very laborious techniques. Therefore, the practical problems associated with all these determinations on a large scale are driving the search for new cheap, widely applicable clinical biomarkers.

Rheology is based on the evaluation of flow and deformation responses of a substance when subjected to stress [5]. Specifically, viscosity is the resistance of a fluid to a deforming force by either shear or tensile stress. It provides information about the microstructure and properties of a fluid or soft material and is usually shear-rate dependent in non-Newtonian fluids [6]. Therefore, taking into account that differences in chemical and molecular composition of biological samples affected viscosity, it is thought that this property could be linked to clinical risk factors.

In the case of blood, viscosity depends on haematocrit, erythrocytes deformability and plasma viscosity, which in turn depends mostly on its protein profile. Since different pathologies (from rheumatoid arthritis to angina pectoris) are associated with modifications in these parameters, blood or plasma viscosity–depending on the case- have been proposed as a marker for them [7]. In the particular context of cardiometabolic diseases, this measurement has been shown to be significantly increased in subjects with already-diagnosed metabolic syndrome or in old subjects [8], but it has been less studied at previous risk stages. In these studies viscosity was determined with simple viscometers such as capillary [7,9] or cone-plate viscometers [8,10], based on the assumption that plasma was a Newtonian fluid (not depending on flow characteristics) [7]. On the contrary, other authors [11–13] have reported non-Newtonian behaviour of whole blood and the importance of a detailed rheological characterization of this fluid. In particular, the characterization of blood flow behaviour and of the distribution of the wall shear stress in small vessels may help detect cardiovascular diseases and design suitable treatments [14], suggesting the use of a rotational rheometer within a wide range of shear rates. Nevertheless, this kind of rotational rheometer has been scarcely used up to date.

In the case of urine, some studies have examined viscosity or other related parameters in healthy individuals or in certain pathological situations [15–17] as possible early clinical markers. However, all these studies have assumed that urine is a Newtonian fluid, although to the author’s knowledge there is no work justifying this statement. Finally, only one study has explored the potential association between viscosity of biological samples and oxidative status [10]. The authors just cited found an increase in plasma viscosity in subjects with increased oxidative stress (measured as malondialdehyde), but in a very specific population, i.e., subjects with severe obstructive sleep apnea syndrome.

Therefore, the aim of this study was to evaluate whether plasma and urine viscosity determinations in subjects at a primary stage of cardiometabolic risk were associated with clinical risk factors as well as with oxidative status. To this end, a pilot clinical trial (*n* = 20) was conducted in subjects with abdominal obesity. New findings regarding the association between urine viscosity and oxidative status were obtained.

## Methods

### Subjects

Subjects were participating in a study on the effects of grape and pomegranate polyphenols on cardiometabolic risk factors and oxidative status, where viscosity was included as a related measurement. The cardiometabolic criteria for inclusion in the study was abdominal obesity, defined as abdominal diameter > 94 cm for men and > 80 cm for women [2]- although other anthropometric parameters were also measured, as explained below. Besides, the subjects should be apparently healthy; not medicated for cardiometabolic pathologies; and aged between 40 and 60 years. Exclusion criteria were: pregnant or lactating; previously diagnosed with a chronic disease (cardiovascular diseases, inflammatory diseases, diabetes or cancer); any regular pharmaceutical treatment or nutritional supplements; participating in any other dietary intervention study.

This study was approved by the Ethics Subcommittee of the CSIC, Madrid, Spain (2015/12/21) and the Ethics Committee for Clinical Research of the University Hospital Puerta de Hierro-Majadahonda, Majadahonda, Spain (2015/11/23). It was registered in the Clinical Trials database (NCT02710461). The study was conducted between February and June 2016 at the Unit of Human Nutrition of the ICTAN-CSIC. All the subjects signed a written consent form agreeing to participate in the study.

A total of 20 subjects, 10 male and 10 female, participated in the study. Besides the nutritional intervention they later followed, this sample size was considered as appropriate for the evaluation of the association between plasma and urine viscosity with cardiometabolic biomarkers at baseline, based on a previous study defining an association between Body Mass Index (BMI) and plasma viscosity [18], and considering an 80% power and an unilateral alpha value of 0.05. All subjects fulfilled the criteria for abdominal obesity (mean values, 110 ± 9 cm for males, 100 ± 9 for females) and, based on body mass index (BMI) values, 35% were overweight and 40% were obese (mean BMI, 29.7 ± 2.7). Additionally, 50% exhibited a waist-to-hip ratio (WHR) above recommendations (mean values, 0.98 ± 0.09 for males, 0.84 ± 0.13 for females) [19]. Regarding cardiometabolic risk factors [2], 25% of the subjects showed high systolic pressure (< 130 mm Hg), 50% high diastolic pressure (> 85 mm Hg), 15% high triglycerides (> 150 mg/dL), 20% low high-density lipoprotein (HDL)-cholesterol (< 50 mg/dL for women and 40 mg/dL for men), 60% high low-density lipoprotein (LDL)-cholesterol (> 130 mg/dL) and 55% high total cholesterol (> 200 mg/dL). Further details on the subjects have been reported elsewhere[20]. The present study is based on baseline determinations in these subjects, independently of the treatments they followed later as part of the mentioned nutritional intervention study.

### Biological samples

Samples used for the present study conformed to basal values of the clinical study, so the volunteers had not received any supplementation. Fasting venous blood samples were collected in tubes with sodium heparin as anticoagulant, centrifuged, and plasma was recovered. Fasting urine was recovered 0–3 h after arrival at the Unit of Human Nutrition of the ICTAN-CSIC first thing in the morning. Plasma and urine samples were stored at -80°C until analysis.

Subjects followed their normal lifestyle, although seventy-two hours prior to sample collection, they were required to refrain from consumption of polyphenol-rich foods due to the further supplementation they were going to receive; since this was a very punctual modification of their dietary habits, it may be excluded that it affected the biochemical measurements carried out in the samples, or the viscosity determinations.

### Viscosity measurements

Viscosity was measured in a Kinexus pro rheometer (Malvern Instruments Ltd., Worcestershire, UK) equipped with a cone-plate of 60 mm diameter, 1˚ angle and a gap of 0.030 mm. One millilitre of urine or 100 μL of plasma (1:15 dilution) was placed with a pipette on a pre-heated plate (37°C). Temperature was controlled to within 0.1°C by Peltier elements in the lower plate. A temperature cover was used to maintain the samples at the specified temperature (37°C) and prevent evaporation. To homogenize mechanical equilibrium before measurements, all the samples were put through a pre-shearing test for one min at 100 s^–1^, 37°C. Flow curves were then plotted as a function of shear rate ranging from 1000 to 50 s^–1^ for urine samples and from 10000 to 1000 s^–1^ for plasma samples. At least three flow curves were made for each individual choosing the shear rates more convenient for each biological sample to obtain proper correlations of flow curves. Viscosities were taken at nine different descending shear rates by decade for statistical analysis of the flow curves.

### Biochemical determinations

Regarding cardiometabolic biomarkers, blood glucose was determined using a glucometer from Abbott (Chicago, IL, USA). Plasma insulin was determined with an ELISA kit (Millipore, MA, USA). Lipid profile was measured by standard automated methodologies.

For oxidative status evaluation, Plasma and urine uric acid were determined using an enzymatic-colorimetric kit (Spin React, San Esteve de Bas, Girona, Spain). Blood and urine antioxidant capacity were estimated using the ferric reducing ability of plasma (FRAP) and 2,2’-azinobis(3-ethylbenzothiazoline-sulfonic acid (ABTS) assays [21–23]. The Folin-Ciocalteu assay was used after solid phase extraction to evaluate the polyphenol content of urine [24].

All urine measurements were normalized using creatinine concentration, measured via the colorimetric Jaffe reaction. The values recorded in these determinations, detailed elsewhere [20], were used to evaluate correlations with viscosity measurements.

### Statistical analysis

Data were analysed with the statistical SPSS IBM 19 package for Windows. First, normal data distribution was evaluated and data on urine viscosity at 398 and 1000 s^–1^ were log transformed. For comparing the values obtained at the different shear rates, one-way ANOVA followed by post-hoc Tukey test were applied. Pearson correlation coefficients were calculated to examine the relationship between urine and plasma viscosities and cardiometabolic risk factors. In this way the variables correlating with plasma or urine viscosity were identified and then used in categorical principal components analysis (CATPCA). Additionally, and in order to discard the possible confounding role of age and central obesity on oxidative stress results, Factorial Principal Component Analysis was performed including viscosity and these variables. Similarly, Partial Correlation Analysis adjusted by age and central obesity measures was performed. These tests were only carried out for urine values since in blood no association between these variables was found.

### Results

**Fig 1:**
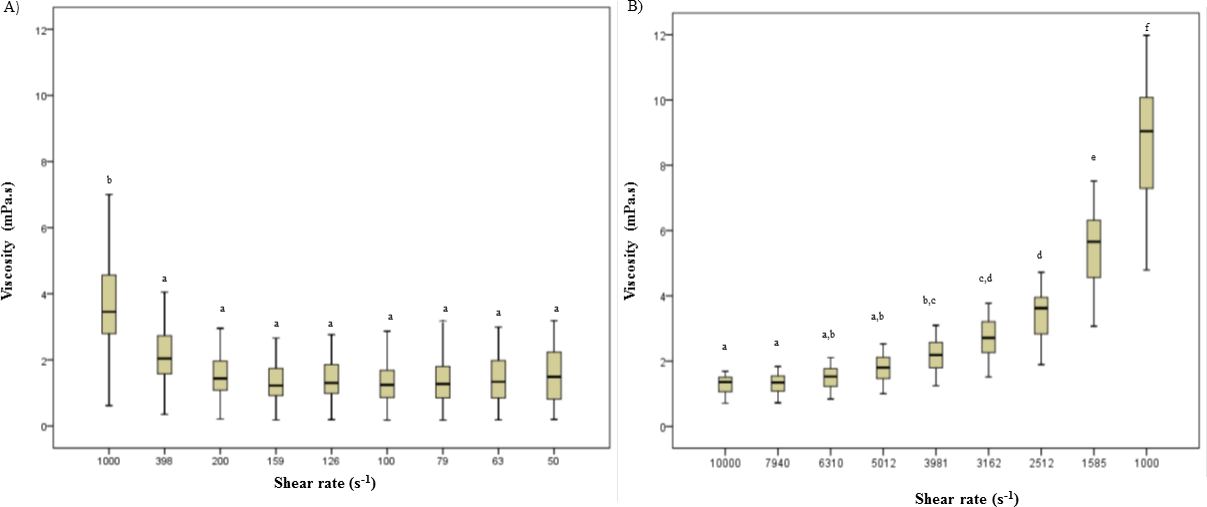
Viscosity values (mPa s) of human urine and plasma from subjects with abdominal obesity at different shear rates (s–1) selected from flow curves; (A) urine; (B) plasma. Different superscript letter indicate significant differences (P < 0.05) between shear rates.

Fig 1 shows the median viscosity and the upper and lower whiskers values in urine (A) and plasma (B) from subjects with abdominal obesity. Different shear ranges were taken for each fluid (10000–1000 s^–1^ in plasma and 1000–50 s^–1^ in urine) given the probable differences in biochemical composition of the two fluids. Indeed, viscosity values in plsma were higher than in urine. Regarding values at most shear rates, no significant differences were observed in urine, with only those at 1000 s^–1^ being higher than the rest (*p*< 0.05); in contrast, significant differences between most shear rates (*p*< 0.05) were found in plasma. In a higher shear rate range, plasma samples (Fig 1B) showed well-defined non-Newtonian shear-thinning, with viscosity values decreasing from 8.68 ± 1.77 to 1.28 ± 0.270 mPa s with increasing shear rate from 1000 to 10000 s^–1^. On the contrary, in a lower shear rate range, urine viscosity trends to increase with increasing shear rate (Fig 1A) from 1.38 ± 0.860 up to 4.07 ± 3. 05 mPa s between 100 and 1000 s^–1^, although values at the lowest shear rate (50 s^–1^) were intermediate and close to those obtained at 200 s^–1^ (1.64 ± 1.09 and 1.62 ± 1.09 mPa s, respectively). Individual viscosity values for all the subjects are shown in S1 Table and S2 Table.

Correlation coefficients between urine and plasma viscosities and cardiometabolic risk factors are shown in Tables 1 and 2 respectively; individual values for cardiometabolic risk factors are shown in S3 Table, S4 Table and S5 Table. At eight of the selected shear rates, urine viscosity correlated significantly either with age or with antioxidant capacity (TEAC) (Table 1). However, age did not correlate with urine viscosity at 100 and 50 s^–1^ respectively. On the other hand, at both 100 and 50 s^–1^, urine viscosity also correlated significantly with polyphenols. These associations were kept when Partial Correlation Analysis adjusted by age and central obesity measures (abdominal perimeter, WHR, WST) was performed. On the contrary, the observed significant association between urine viscosity and uric acid (at shear rate of 50) was lost when Partial Correlation Analysis was adjusted by age, being therefore mediated by this parameter. For its part, plasma viscosity was only found to correlate with glucose (Table 2).

**Table 1:** Correlations (*R* and *p* values) among diverse cardiometabolic risk factors and viscosity measurements at different shear rates (s^-1^) in human urine.

					Shear rate (s^–1^)				
	1000	398	200	159	126	100	79	63	50
Anthropometry									
Sex	0.202	0.181	0.250	0.155	0.205	0.101	0.038	0.026	0.070
Age	**-0.447 (0.048)**	**-0.481 (0.032)**	**-0.530 (0.016)**	**-0.559 (0.007)**	**-0.557 (0.011)**	-0.585 (0.070)	**-0.584 (0.007)**	**-0.577 (0.008)**	**-0.562 (0.010)**
Abdominal perimeter	-0.056	-0.025	-0.101	-0.031	-0.075	0.000	0.045	0.095	0.125
BMI	-0.068	-0.046	-0.012	0.022	-0.003	0.027	0.047	0.074	0.087
WHR	-0.190	-0.179	-0.353	-0.208	-0.285	-0.117	-0.015	0.081	0.151
WSR	-0.195	-0.174	-0.277	-0.179	-0.238	-0.127	-0.053	0.019	0.069
Biochemistry									
Uric Acid	-0.450	-0.071	0.073	0.003	0.038	0.413	0.086	0.122	0.467
Polyphenols	0.190	0.194	0.364	0.336	0.349	**0.677 (0.001)**	0.274	0.242	**0.701 (0.001)**
Antioxidant capacity (FRAP)	0.198	0.181	0.184	0.149	0.160	0.113	0.116	0.099	0.132
Antioxidant capacity (TEAC)	**0.693 (0.001)**	**0.668 (0.001)**	**0.736 (0.000)**	**0.664 (0.010)**	**0.702 (0.010)**	**0.617 (0.040)**	**0.546 (0.013)**	**0.470 (0.037)**	0.478

Significant correlations (*p* ≤ 0.05) are given in boldface. BMI, body mass index; WHR, waist-to-hip ratio; WSR, waist-to-stature ratio; FRAP, ferric reducing antioxidant power; TEAC, trolox equivalent antioxidant.

**Table 2:** Correlations (*R* and *p* values) among diverse cardiometabolic risk factors and viscosity measurements at different shear rates (s^-1^) in human plasma.

					Shear rate (s^–1^)				
	10000	7944	6310	5012	3981	3162	2512	1585	1000
*Anthropometry*									
Sex	0.123	0.118	0.134	0.130	0.128	0.130	0.074	0.091	0.068
Age	0.283	0.260	0.300	0.298	0.301	0.325	0.341	0.336	0.325
Abdominal perimeter	-0.269	-0.257	-0.261	-0.243	-0.232	-0.243	-0.206	-0.240	-0.241
BMI	0.071	0.088	0.058	0.072	0.077	0.066	0.095	0.075	0.087
WHR	-0.125	-0.096	-0.085	-0.065	-0.054	-0.063	-0.074	-0.134	-0.140
WSR	0.103	0.118	0.122	0.140	0.151	0.143	0.139	0.083	0.087
Systolic pressure	0.186	0.210	0.223	0.224	0.205	0.191	0.215	0.199	0.224
Diastolic pressure	0.047	0.036	0.007	0.014	0.001	-0.025	0.015	0.025	0.061
*Biochemistry*									
Triglycerides	0.326	0.361	0.363	0.380	0.366	0.354	0.392	0.417	0.421
HDL-cholesterol	-0.005	-0.001	0.018	0.005	0.021	0.040	-0.005	0.007	0.034
LDL-cholesterol	0.070	0.063	0.087	0.076	0.075	0.071	0.014	0.032	0.023
Total cholesterol	0.161	0.163	0.197	0.186	0.190	0.193	0.134	0.165	0.173
Glucose	**0.639 (0.002)**	**0.664 (0.001)**	**0.669 (0.001)**	**0667 (0.001)**	**0.673 (0.001)**	**0.680 (0.001)**	**0.669 (0.001)**	**0.654 (0.002)**	**0.672 (0.001)**
Insuline	0.210	0.267	0.270	0.292	0.312	0.306	0.362	0.335	0.340
HOMA index	0.269	0.328	0.331	0.352	0.372	0.369	0.421	0.393	0.406
HOMA-beta index	-0.112	-0.048	-0.042	-0.026	-0.005	-0.014	0.035	0.012	0.012
Quicky index	-0.158	-0.225	-0.231	-0.253	-0.265	-0.251	-0.296	-0.271	-0.255
Uric Acid	0.186	0.227	0.211	0.215	0.221	0.218	0.251	0.245	0.241
Antioxidant capacity (FRAP)	0.187	0.229	0.214	0.218	0.224	0.221	0.253	0.247	0.243
Antioxidant capacity (TEAC)	-0.376	-0.374	-0.363	-0.375	-0.364	-0.369	-0.331	-0.356	-0.369

Significant correlations (*p* ≤ 0.05) are given in boldface. BMI, body mass index; WHR, waist-to-hip ratio; WSR, waist-to-stature ratio; HOMA, homeostatic model assessment; FRAP, ferric reducing antioxidant power; TEAC, trolox equivalent antioxidant capacity.

**Fig 2:**
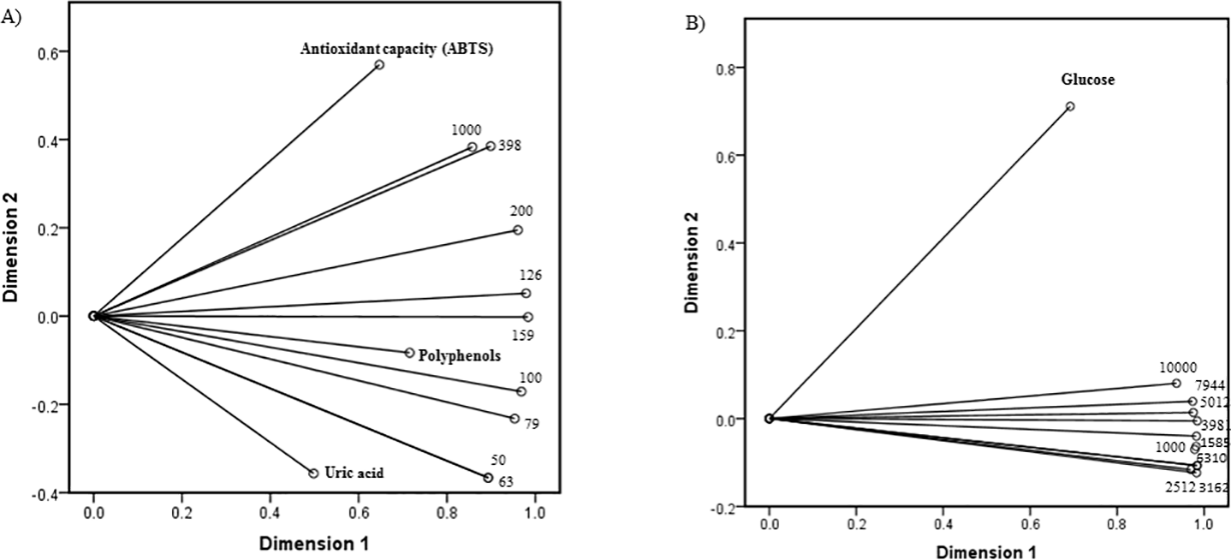
Categorical principal components analysis (CATPCA) in samples from subjects with abdominal obesity; (A) Biplot-CATPCA urine; (B) Biplot-CATPCA-Plasma. Numbers are representing different shear rates used to determine viscosity at each biological fluid.

CATPCA accounts for almost 85% of the variance of the variables under analysis in the case of urine, and for 92% in the case of plasma, indicating the goodness of the fits of the components for both biological fluids (Table 3). Also, the first two dimensions for each cardiometabolic risk factor are shown in urine (Fig 2A) and plasma (Fig 2B). Both biplots overlap the shear rates (indicated by points) and the component loadings (of each item, indicated by vectors) form CATPCA along both dimensions. The coordinates of the end point of each vector are given by the loadings of each variable on the first and second components. Long vectors indicate good fit. The cosines of the angles between the vectors equal the correlations between the quantified variables. It can be observed that in scatterplots for both biological fluids, dimension 1 is able to capture more of the variance than dimension 2. In the case of urine (Fig 2A), viscosity measurements at different shear rates are clustered in three groups (low, intermediate and high shear rates) associated with uric acid, polyphenols and antioxidant capacity respectively. On the other hand, all viscosity values are clustered together (Fig 2B).

**Table 3:** Summary of categorical principal components analysis. (CATPCA) for human urine and plasma.

**Urine CATPCA model**			
**Dimension**	**Cronbach’s alpha**	**Variance**	** **
** **	** **	**Total (eigenvalue)**	**% of variance**
1	0.970	9.01	75.1
2	0.138	1.15	9.54
Total	0.984^a^	10.2	84.6
**Plasma CATPCA model**			
1	0.985	11.1	85.2
2	-0.069	0.940	7.23
Total	0.993^a^	12.0	92.4

^a^ Cronbach’s alpha means is based on the mean of the eigenvalue.

## Discussion

The present study aimed to evaluate the usefulness of viscosity measurements in biological fluids as a way to integrate several parameters of cardiometabolic risk and associated altered oxidative status in subjects in the early stages of cardiometabolic risk. This might serve as a strategy in prior screening of subjects or in large-scale population studies, in order to decide in which cases detailed biochemical determinations should be adopted later on. To this end, a pilot study (*n* = 20) was conducted in subjects with abdominal obesity, determining urine and plasma viscosity with a rotational rheometer which makes it possible to characterize the flow over a wide shear rate range (from 50 to 1000 s^-1^ and 1000 to 10000 s^-1^ respectively). Overall, the viscosity values were in the same range as previously reported in other studies [13,16]. Similarly, some of the results agree with previous ones, such as the lack of association between plasma viscosity and age or gender [7].

Nevertheless, in this study, unlike previous ones based on the use of simple viscometers, a rheometer with a range of shear rates was applied, with the object of assessing the potential differential information provided by different shear rates in subjects in the early stages of cardiometabolic disorders. Interestingly, a previous study on the association of plasma viscosity and lipid profile also observed that specific shear rates were associated with different physiological characteristics [25]. The present results indicate that while values in plasma viscosity decreased, urine viscosity tended to increase as shear rates increased.

Whole blood is well characterized in the literature in terms of steady shear rheology, and flow curves have been published identifying several erythrocyte characteristics as the main contributing factors to its viscosity [13,26,27]. For its part, plasma is a highly concentrated protein solution, and therefore weak protein-protein interactions could play an important role in the viscosity of this fluid. Despite promising results on the clinical relevance of plasma viscosity, this fluid has been less studied than blood viscosity [7]. Indeed, it has been suggested that plasma viscosity may be more sensitive than blood viscosity to changes in the plasma proteins associated with cardiovascular disease risk and mortality, and hence would be more valid as a clinical marker of these situations [9]. Therefore, plasma viscosity rather than blood viscosity was evaluated in the present study. Nevertheless, both simple regression and CATPCA plots (Table 2 and Fig 2B) indicated that at all the tested shear rates plasma viscosity values were positively associated with blood glucose but not with any of the other parameters evaluated. There are easy and cheap quantitative methods to determine blood glucose, so given that this was the only association found, results from the present study indicate that viscosity measurement in plasma is not clinically relevant to cardiometabolic risk or oxidative status in subjects with abdominal obesity. This conflicts with a previous study, in which the authors found associations between plasma viscosity and all components of metabolic syndrome but glucose [8] although in this case the subjects already exhibited metabolic syndrome.

On the other hand, several significant associations were detected between viscosity measurements in urine, although these are not associated with cardiometabolic risk, and oxidative status (Fig 2A). In particular, CATPCA plots showed that the lowest shear rates tested (50 and 63 s^–1^) were associated with urine uric acid, the intermediate rates (100 and 156 s^–1^) with urine polyphenols and the highest (398 and 1000 s^–1^) with urine antioxidant capacity, as determined by TEAC assay. Inverse associations have been reported between increased antioxidant capacity in biological fluids and several cardiometabolic risk factors [28,29]. Nevertheless, *in vivo* antioxidant capacity is known to be derived from different constituents: a) polyphenols, phytochemicals increased levels of which in urine have also been associated with health benefits [30,31], b) uric acid, a well-described marker of cardiovascular risk [32]. Indeed, antioxidant capacity values are commonly reported after subtracting the contribution of uric acid, i.e., reporting only that of polyphenols [33]. Therefore, the results of this pilot study suggest that urine viscosity at several shear rates may be used as a first screening tool for different components of oxidative status. Nevertheless, the previously mentioned potential confounding role of age regarding the association between urine viscosity and uric acid should not be disregarded. Therefore, these results should be confirmed in a wider population, since at this time there is a scarcity of human urine viscosity measurements.

In conclusion, a pilot study was conducted in subjects with abdominal obesity to evaluate the usefulness of viscosity measurements in urine and plasma as indicators of cardiometabolic risk and oxidative status in this population. Plasma viscosity did not seem to be relevant in this connection, but significant associations were detected between urine viscosity and oxidative status. In particular, urine viscosity measured at different shear rates (from 50 to 1000 s^–1^) was selectively associated with urine antioxidant capacity, polyphenols and uric acid. This indicates a potential for urine viscosity to be used as a complimentary marker in the evaluation of oxidative status, something that needs to be confirmed in wider populations.

## Supporting information

S1 Table Raw data for plasma viscosity values (mPa). (DOC)

S2 Table Raw data for urine viscosity values (mPa), after creatinin correction. (DOC)

S3 Table Raw data for demography and anthropometric measurements. (DOC)

S4 Table Raw data for cardiometabolic markers. (DOC)

S5 Table Raw data for oxidative stress markers. (DOC)
