# Supplementary material for: Association of plasma and urine viscosity with cardiometabolic risk factors and oxidative status. A pilot study in subjects with abdominal obesity
Source: PLoS One. 2018 Oct 9;13(10):e0204075. doi: 10.1371/journal.pone.0204075 (PMC6177142; doi:10.1371/journal.pone.0204075)
Supplement: S1 Table — (DOC) [file pone.0204075.s001.doc]

**S1 Table**. Raw data for plasma viscosity values (mPa)

| Subject | Shear rate (s^-1^) | | | | | | | | | | |
| --- | --- | --- | --- | --- | --- | --- | --- | --- | --- | --- | --- |
|  | 10,000 | 7,940 | 6,310 | 5,012 | 3,981 | 3,162 | 2,512 | 1,995 | 1,585 | 1,259 | 1,000 |
| 1 | 1.52 | 1.42 | 1.58 | 1.84 | 2.24 | 2.78 | 3.51 | 4.41 | 5.47 | 7.01 | 8.90 |
| 2 | 1.47 | 1.51 | 1.75 | 2.10 | 2.55 | 3.20 | 3.92 | 4.90 | 6.16 | 8.09 | 10.22 |
| 3 | 1.52 | 1.56 | 1.76 | 2.07 | 2.53 | 3.11 | 3.74 | 4.66 | 5.91 | 7.37 | 9.26 |
| 4 | 1.51 | 1.55 | 1.79 | 2.14 | 2.61 | 3.23 | 4.02 | 5.01 | 6.43 | 8.00 | 10.05 |
| 5 | 1.06 | 1.10 | 1.25 | 1.48 | 1.81 | 2.24 | 2.81 | 3.56 | 4.51 | 5.76 | 7.27 |
| 6 | 1.49 | 1.54 | 1.79 | 2.15 | 2.60 | 3.23 | 3.99 | 5.01 | 6.35 | 8.19 | 10.11 |
| 7 | 1.35 | 1.32 | 1.49 | 1.75 | 2.14 | 2.61 | 3.24 | 4.10 | 5.26 | 6.60 | 8.44 |
| 8 | 1.51 | 1.60 | 1.83 | 2.19 | 2.69 | 3.36 | 4.09 | 5.25 | 6.61 | 8.36 | 10.56 |
| 9 | 1.55 | 1.64 | 1.98 | 2.33 | 2.79 | 3.48 | 4.32 | 5.47 | 6.65 | 8.28 | 10.32 |
| 10 | 1.69 | 1.84 | 2.11 | 2.53 | 3.10 | 3.78 | 4.72 | 6.00 | 7.52 | 9.46 | 11.99 |
| 11 | 1.07 | 1.07 | 1.21 | 1.46 | 1.78 | 2.25 | 2.68 | 3.35 | 4.25 | 5.24 | 6.63 |
| 12 | 1.02 | 1.05 | 1.20 | 1.44 | 1.79 | 2.28 | 2.87 | 3.74 | 4.87 | 6.78 | 8.09 |
| 13 | 1.26 | 1.29 | 1.47 | 1.77 | 2.14 | 2.65 | 3.75 | 4.78 | 6.29 | 7.32 | 9.68 |
| 14 | 1.12 | 1.14 | 1.28 | 1.52 | 1.86 | 2.29 | 2.88 | 3.67 | 4.61 | 5.72 | 7.32 |
| 15 | 0.96 | 1.01 | 1.13 | 1.34 | 1.63 | 2.05 | 2.59 | 3.37 | 4.10 | 5.07 | 6.49 |
| 16 | 0.87 | 0.93 | 1.03 | 1.26 | 1.50 | 1.84 | 2.35 | 2.95 | 3.85 | 4.97 | 6.34 |
| 17 | 1.41 | 1.45 | 1.64 | 1.98 | 2.43 | 2.96 | 3.76 | 4.73 | 5.99 | 7.50 | 9.19 |
| 18 | 0.71 | 0.73 | 0.84 | 1.00 | 1.25 | 1.52 | 1.89 | 2.30 | 3.07 | 3.74 | 4.79 |
| 19 | 1.07 | 1.15 | 1.35 | 1.65 | 2.05 | 2.60 | 3.29 | 4.11 | 5.30 | 6.80 | 8.50 |
| 20 | 1.37 | 1.37 | 1.56 | 1.87 | 2.31 | 2.90 | 3.85 | 4.65 | 5.85 | 7.46 | 9.47 |
