# Supplementary material for: Association of plasma and urine viscosity with cardiometabolic risk factors and oxidative status. A pilot study in subjects with abdominal obesity
Source: PLoS One. 2018 Oct 9;13(10):e0204075. doi: 10.1371/journal.pone.0204075 (PMC6177142; doi:10.1371/journal.pone.0204075)
Supplement: S2 Table — (DOC) [file pone.0204075.s002.doc]

**S2 Table**. Raw data for urine viscosity values (mPa), after creatinin correction

| Subject | Shear rate (s^-1^) | | | | | | | | |
| --- | --- | --- | --- | --- | --- | --- | --- | --- | --- |
|  | 1,000 | 398 | 200 | 159 | 126 | 100 | 79 | 63 | 50 |
| 1 | 2.19 | 1.26 | 1.10 | 1.01 | 1.08 | 1.03 | 1.09 | 1.15 | 1.25 |
| 2 | 2.86 | 1.84 | 1.56 | 1.60 | 1.56 | 1.57 | 1.73 | 1.94 | 2.22 |
| 3 | 3.69 | 2.25 | 1.86 | 1.87 | 1.85 | 1.95 | 2.10 | 2.32 | 2.63 |
| 4 | 0.62 | 0.35 | 0.21 | 0.18 | 0.19 | 0.18 | 0.18 | 0.19 | 0.20 |
| 5 | 14.87 | 8.35 | 5.19 | 3.92 | 4.45 | 3.49 | 3.18 | 2.99 | 2.96 |
| 6 | 4.54 | 2.34 | 1.52 | 1.38 | 1.41 | 1.41 | 1.49 | 1.66 | 1.88 |
| 7 | 6.97 | 4.05 | 2.96 | 2.66 | 2.77 | 2.65 | 2.76 | 2.86 | 3.19 |
| 8 | 2.93 | 1.62 | 1.07 | 0.75 | 0.91 | 0.66 | 0.60 | 0.57 | 0.57 |
| 9 | 3.87 | 2.20 | 1.31 | 1.15 | 1.19 | 1.14 | 1.17 | 1.25 | 1.36 |
| 10 | 4.27 | 2.71 | 2.42 | 2.62 | 2.48 | 2.87 | 3.24 | 3.78 | 4.45 |
| 11 | 7.01 | 3.79 | 2.32 | 1.68 | 1.98 | 1.43 | 1.24 | 1.09 | 0.99 |
| 12 | 1.07 | 0.64 | 0.40 | 0.36 | 0.37 | 0.37 | 0.39 | 0.42 | 0.47 |
| 13 | 4.41 | 2.36 | 1.55 | 1.23 | 1.36 | 1.14 | 1.08 | 1.06 | 1.10 |
| 14 | 4.59 | 2.75 | 1.92 | 1.63 | 1.75 | 1.56 | 1.57 | 1.65 | 1.78 |
| 15 | 2.72 | 1.54 | 1.12 | 1.01 | 1.05 | 1.01 | 1.04 | 1.13 | 1.26 |
| 16 | 2.89 | 1.65 | 1.26 | 1.21 | 1.21 | 1.26 | 1.36 | 1.53 | 1.75 |
| 17 | 4.84 | 2.76 | 2.02 | 1.81 | 1.87 | 1.80 | 1.88 | 2.02 | 2.25 |
| 18 | 2.97 | 1.89 | 1.37 | 1.21 | 1.24 | 1.23 | 1.30 | 1.43 | 1.62 |
| 19 | 0.99 | 0.54 | 0.30 | 0.24 | 0.27 | 0.22 | 0.21 | 0.21 | 0.22 |
| 20 | 3.22 | 1.76 | 1.07 | 0.83 | 0.93 | 0.71 | 0.66 | 0.64 | 0.64 |
