# Supplementary material for: Association of plasma and urine viscosity with cardiometabolic risk factors and oxidative status. A pilot study in subjects with abdominal obesity
Source: PLoS One. 2018 Oct 9;13(10):e0204075. doi: 10.1371/journal.pone.0204075 (PMC6177142; doi:10.1371/journal.pone.0204075)
Supplement: S3 Table — (DOC) [file pone.0204075.s003.doc]

**S3 Table**. Raw data for demography and anthropometric measurements

| Subject | Sex^1^ | Age | Abdominal perimeter (cm) | WHR | BMI | WSR |
| --- | --- | --- | --- | --- | --- | --- |
| 1 | 1 | 55 | 102 | 0.95 | 24.94 | 0.54 |
| 2 | 1 | 43 | 115 | 0.95 | 34.34 | 0.65 |
| 3 | 1 | 47 | 107 | 1.00 | 28.43 | 0.58 |
| 4 | 2 | 51 | 99 | 0.79 | 33.53 | 0.57 |
| 5 | 2 | 41 | 100 | 0.69 | 29.72 | 0.48 |
| 6 | 2 | 50 | 103 | 0.88 | 28.76 | 0.58 |
| 7 | 2 | 47 | 92 | 0.89 | 25.96 | 0.53 |
| 8 | 2 | 55 | 89 | 0.86 | 23.99 | 0.51 |
| 9 | 2 | 61 | 95 | 0.82 | 27.44 | 0.56 |
| 10 | 1 | 41 | 109 | 1.02 | 31.57 | 0.59 |
| 11 | 2 | 45 | 101 | 0.83 | 30.45 | 0.52 |
| 12 | 2 | 53 | 99 | 0.83 | 24.62 | 0.52 |
| 13 | 1 | 49 | 104 | 0.90 | 23.93 | 0.48 |
| 14 | 2 | 40 | 104 | 0.82 | 29.47 | 0.57 |
| 15 | 1 | 39 | 107 | 1.02 | 29.94 | 0.56 |
| 16 | 1 | 45 | 107 | 1.04 | 29.97 | 0.59 |
| 17 | 2 | 50 | 117 | 1.01 | 34.95 | 0.71 |
| 18 | 1 | 48 | 122 | 0.90 | 35.42 | 0.62 |
| 19 | 1 | 57 | 109 | 0.99 | 31.15 | 0.61 |
| 20 | 1 | 52 | 116 | 1.00 | 36.11 | 0.67 |

WHR, waist-to-hip ratio; BMI, body mass index; WSR, waist-to-stature ratio.

^1^ 1, male; 2, female.
