# Supplementary material for: Association of plasma and urine viscosity with cardiometabolic risk factors and oxidative status. A pilot study in subjects with abdominal obesity
Source: PLoS One. 2018 Oct 9;13(10):e0204075. doi: 10.1371/journal.pone.0204075 (PMC6177142; doi:10.1371/journal.pone.0204075)
Supplement: S4 Table — (DOC) [file pone.0204075.s004.doc]

**S4 Table**. Raw data for cardiometabolic markers

|  | Systolic blood  pressure (mmHg) | Dyastolic blood pressure (mmHg) | Triglycerides (mg/dL) | HDL cholesterol (mg/dL) | Total colesterol (mg/dL) | LDL colesterol (mg/dL) | Glucosa (mg/dL) | Insulin (μU/mL) | HOMA-IR | HOMA-b | QUICKI |
| --- | --- | --- | --- | --- | --- | --- | --- | --- | --- | --- | --- |
| 1 | 115 | 81 | 105 | 43 | 203 | 139 | 103 | 2.96 | 0.75 | 24.20 | 4.15 |
| 2 | 131 | 94 | 194 | 45 | 236 | 153 | 102 | 4.29 | 1.08 | 32.85 | 3.62 |
| 3 | 130 | 87 | 116 | 44 | 223 | 156 | 99 | 4.77 | 1.17 | 39.90 | 3.50 |
| 4 | 124 | 82 | 82 | 64 | 178 | 98 | 93 | 4.83 | 1.11 | 45.72 | 3.47 |
| 5 | 110 | 83 | 68 | 90 | 220 | 116 | 95 | 4.69 | 1.10 | 76.81 | 3.42 |
| 6 | 134 | 92 | 138 | 48 | 227 | 151 | 100 | 3.79 | 0.94 | 34.10 | 3.74 |
| 7 | 128 | 97 | 47 | 67 | 217 | 141 | 86 | 3.68 | 0.78 | 47.33 | 3.73 |
| 8 | 109 | 81 | 73 | 74 | 239 | 151 | 99 | 4.00 | 0.97 | 37.91 | 3.66 |
| 9 | 129 | 75 | 100 | 62 | 229 | 147 | 99 | 3.61 | 0.89 | 36.05 | 3.79 |
| 10 | 127 | 86 | 177 | 50 | 198 | 112 | 104 | 13.18 | 3.37 | 98.87 | 2.94 |
| 11 | 100 | 69 | 85 | 57 | 192 | 119 | 93 | 3.42 | 0.79 | 41.01 | 3.84 |
| 12 | 113 | 78 | 68 | 87 | 234 | 132 | 89 | 2.42 | 0.53 | 31.16 | 4.56 |
| 13 | 123 | 89 | 186 | 35 | 205 | 132 | 96 | 4.87 | 1.15 | 43.84 | 3.47 |
| 14 | 117 | 86 | 73 | 57 | 162 | 90 | 83 | 3.19 | 0.65 | 49.98 | 3.92 |
| 15 | 115 | 78 | 42 | 46 | 150 | 104 | 88 | 3.16 | 0.69 | 43.80 | 3.95 |
| 16 | 142 | 107 | 128 | 27 | 156 | 103 | 94 | 4.41 | 1.02 | 42.86 | 3.55 |
| 17 | 108 | 85 | 100 | 38 | 193 | 134 | 95 | 4.21 | 0.98 | 38.91 | 3.61 |
| 18 | 123 | 83 | 109 | 48 | 252 | 182 | 88 | 5.05 | 1.10 | 58.63 | 3.40 |
| 19 | 117 | 75 | 74 | 49 | 194 | 130 | 99 | 9.15 | 2.24 | 78.41 | 3.06 |
| 20 | 130 | 92 | 66 | 59 | 150 | 78 | 93 | 15.68 | 3.60 | 125.41 | 2.87 |

HOMA-IR, homeostatic model assessment for insulin resistance; HOMA-b, homeostatic model assessment for beta-cell function; QUICKI, quantitative insulin sensitivity check index.
