# Supplementary material for: Association of plasma and urine viscosity with cardiometabolic risk factors and oxidative status. A pilot study in subjects with abdominal obesity
Source: PLoS One. 2018 Oct 9;13(10):e0204075. doi: 10.1371/journal.pone.0204075 (PMC6177142; doi:10.1371/journal.pone.0204075)
Supplement: S5 Table — (DOC) [file pone.0204075.s005.doc]

**S5 Table**. Raw data for oxidative stress markers

|  | Plasma measurements | | | Urine measurements | | | |
| --- | --- | --- | --- | --- | --- | --- | --- |
| 1 | Uric acid (mg/dL) | FRAP  (μmol Trolox/L) | TEAC  (μmol Trolox/L) | Uric acid  (mg/g creatinin) | Polyphenols (mg/g creatinin) | FRAP  (μmol Trolox/  g creatinin) | TEAC  (μmol Trolox/  g creatinin) |
| 2 | 5.49 | 326.79 | 2,542.67 | 198.26 | 188.8 | 79.64 | 2,493.34 |
| 3 | 4.74 | 283.68 | 1,933.08 | 506.44 | 112.62 | 471.75 | 1,199.30 |
| 4 | 11.44 | 680.50 | 2162.3 | 289.27 | 196.8 | 279.77 | 4,166.14 |
| 5 | 2.91 | 173.01 | 1,789.88 | 7.05 | 56.34 | 361.99 | 189.51 |
| 6 | 3.05 | 181.25 | 2,891.12 | 649.96 | 225.87 | 1,316.57 | 23,182.92 |
| 7 | 3.81 | 226.83 | 1,782.47 | 1,117.42 | 278.24 | 3,143.66 | 15,310.93 |
| 8 | 2.94 | 174.66 | 2,224.82 | 431.91 | 155.44 | 3,241.07 | 9,087.64 |
| 9 | 3.88 | 230.68 | 2,004.58 | 305.93 | 128.8 | 2,606.80 | 6,873.32 |
| 10 | 4.53 | 269.67 | 2,420.23 | 345.81 | 148.61 | 1,417.31 | 5,111.84 |
| 11 | 14.56 | 866.14 | 2,431.74 | 917.45 | 248.01 | 652.59 | 4,584.52 |
| 12 | 4.82 | 286.70 | 2,230.63 | 401.22 | 136.55 | 1,700.15 | 5,582.41 |
| 13 | 5.06 | 300.98 | 2,210.49 | 175.39 | 70.05 | 745.99 | 3,536.21 |
| 14 | 7.51 | 447.07 | 2,333.62 | 498.96 | 104.75 | 1,088.69 | 5,773.28 |
| 15 | 1.78 | 106.18 | 1,748.77 | 337.12 | 103.02 | 1,879.18 | 9,637.59 |
| 16 | 8.45 | 502.55 | 2,621.2 | 321.4 | 172.53 | 2,130.56 | 7,195.96 |
| 17 | 4.77 | 283.95 | 2,079.33 | 1,412.60 | 107.13 | 3,362.58 | 4,916.96 |
| 18 | 4.52 | 269.12 | 2,371.34 | 581.9 | 172.56 | 2,703.90 | 10,113.99 |
| 19 | 5.19 | 308.67 | 2,909.76 | 50.88 | 171.83 | 3,906.21 | 13,397.04 |
| 20 | 4.96 | 294.94 | 2,583.4 | 111.61 | 66.31 | 3,883.59 | 1,011.73 |

FRAP, ferric reducing antioxidant power; TEAC, trolox equivalent antioxidant capacity.
